# Supplementary material for: Flat acoustics with soft gradient-index metasurfaces
Source: Nat Commun. 2019 Jan 11;10:143. doi: 10.1038/s41467-018-07990-5 (PMC6329837; doi:10.1038/s41467-018-07990-5)
Supplement: Supplementary file 1 — Supplementary Information [file 41467_2018_7990_MOESM1_ESM.pdf]

Supplementary document

Flat Acoustics with Soft Gradient-Index Metasurfaces

Yabin Jin[1,*], Raj Kumar[2,*], Olivier Poncelet[1], Olivier Mondain-Monval[2] & Thomas Brunet[1]

[1]*Université de Bordeaux – CNRS – Bordeaux INP, I2M, 33405 Talence, France*

[2]*Université de Bordeaux – CNRS, CRPP, 33600 Pessac, France*

Supplementary note 1: insertion loss of a metasurface

The overall insertion loss of a graded metasurface is not an obvious characteristic to be defined since, by essence, the metasurface strongly modifies the spatial spectrum of the incoming field. The insertion loss can be defined:

1. globally, from the ratio of the total output energy transmitted through the metasurface over the input energy;
2. locally, from the local impedance mismatch of the metasurface with the surrounding propagation media;
3. or specifically, by analysing the amplitude(s) at some specific locations in the transmitted field according to the main expected characteristics of the designed wavefronts, such as the amplitude at the focal point for a metasurface designed for focusing, or the angular variation of the amplitude for a vortex beam.

Moreover, the notion of insertion loss for a metasurface may also depend on how the metasurface is used:

1. directly in contact with the source as in the present work, with the metasurface deposited onto the active face of a piezoelectric transducer (the outer propagating medium is only on one side);
2. or by insertion (the outer propagating medium is on both sides of the metasurface).

Good control or knowledge of the local amplitude of an incoming field or the source is also required to experimentally quantify how the metasurface locally affects the energy transfer or how each spatial spectral component of the shaped fronts is generated.

For these reasons, defining an overall figure characterising insertion loss in this context is not an easy task.

Because gradient metasurfaces sustain the local phase-shifting of wavefields, the local impedance mismatch and the local intrinsic absorption properties are probably the most relevant and immediately understandable features for understanding losses through a metasurface. The following section provides such information on the metasurfaces designed in that work.

Supplementary note 2: local impedance of the soft porous metasurfaces versus porosity

Lossy acoustic-wave propagation in a soft porous material of porosity Φ (here considered spatially homogeneous) can be macroscopically modelled by a complex-valued wavenumber $k(\Phi) = \omega / c(\Phi) - \mathrm{i}\alpha(\Phi)$ (with the $\exp \mathrm{i}\omega t$ convention), where $c(\Phi)$ and $\alpha(\Phi)$ are the speed of sound and the attenuation coefficient, respectively, at porosity Φ and can both be experimentally measured (see the experimental method in Ref. 14 in the main text).

The complex-valued refractive index in the porous material (to water, or the outer medium to the metasurface) is given by $n(\Phi) = n' + \mathrm{i}n'' = k(\Phi) / k_0$, with $k_0 = \omega / c_0$ as the wavenumber in water. The complex-valued impedance of the porous material is defined from its mass density and wave parameters such as $Z(\Phi) = Z' + \mathrm{i}Z'' = \rho(\Phi)\omega / k(\Phi)$, with $\rho(\Phi) = (1-\Phi)\rho_{\Phi=0}$ and $\rho_{\Phi=0}$ as the mass density of the non-porous elastomeric matrix. The mass of the air porosity is neglected. The impedance can also be written in the following form: $Z(\Phi) = (1-\Phi)\rho_{\Phi=0}c_0 / n(\Phi)$.
The relative impedance $Z(\Phi) / Z_0 = (1-\Phi)\rho_{\Phi=0} / \rho_0 n(\Phi)$ of the soft porous material qualifies the impedance matching/mismatching with the outer propagation medium. For the highest porosity considered here ($\Phi_{\mathrm{max}} = 12\%$), representing the most attenuative materials, the imaginary part of the acoustic index n'' is less than 40% of the real part n'. In the initial approximation, we can neglect the impact of n'' on the impedance value to determine the order of magnitude for $Z(\Phi) / Z_0$, noting that the resulting expression of the impedance may be slightly less accurate for the highest porosities/acoustic indices. Considering the real part of n only, the (real-valued) impedance ratio becomes $Z(\Phi) / Z_0 = (1-\Phi)\rho_{\Phi=0} / \rho_0 n'(\Phi)$. This expression does not consider the phase of the actual impedance but provides an estimate of the modulus within less than 10% accuracy when $n'' / n' \leq 40\%$.
The spatial impedance profiles $Z(x) / Z_0 = Z(\Phi(x)) / Z_0$ along the gradient directions $x = X$ or R or θ are plotted in Fig. S1 for the four samples in accordance with Fig. 1 in the main text. The impedance mismatching between the porous silicone rubbers and water obviously enlarges when the porosity increases due to both the decrease in the local mass density and the increase in the real part of the acoustic index (i.e., the decrease in the speed of sound). The general profiles of $Z(x) / Z_0$ are globally inversely proportional to the index profiles $n(x)$ in the samples as shown in Fig. 1 of the main text.

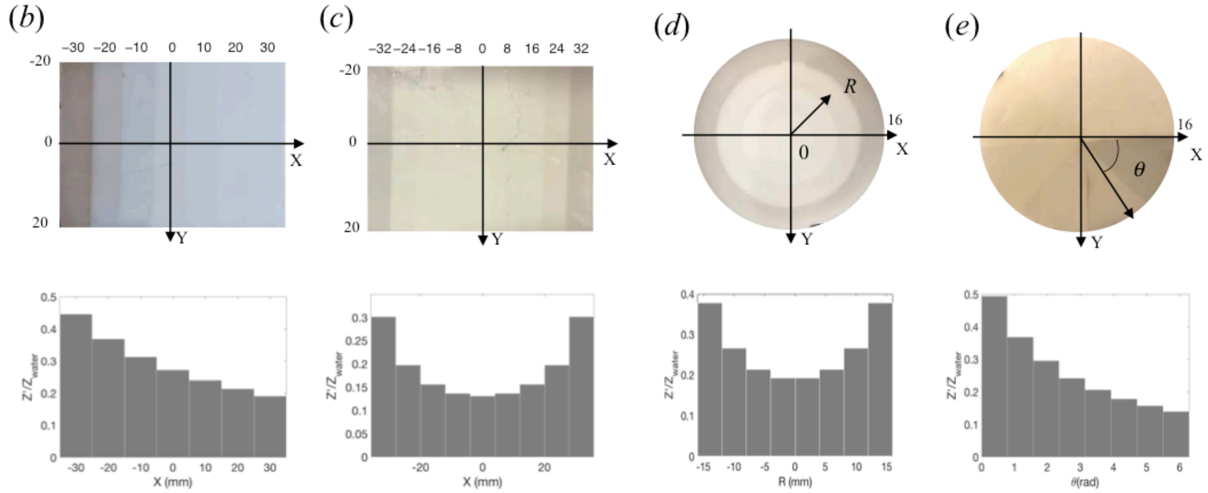

Supplementary Figure 1: Profiles of the relative impedance $Z\left(x\right)/Z_0$ (with Z_0=Z_{water}) for the four studied samples in Fig. 1b-e.

Supplementary note 3: local intrinsic absorption and dispersion of the soft metasurfaces

The acoustic properties of all the different porous units (the speed of sound and the attenuation coefficient) have been measured from porous monodisk samples with thicknesses of 2 mm and 3 mm. Below, we report two examples of the experimental data obtained for porous rubbers of porosity $\Phi = 2.5\%$ and $\Phi = 10\%$. The real and imaginary parts of the dispersive $n\left(\omega;\Phi\right)$ and $Z\left(\omega;\Phi\right)/Z_0$ are plotted in Fig. S2 as a function of the frequency.

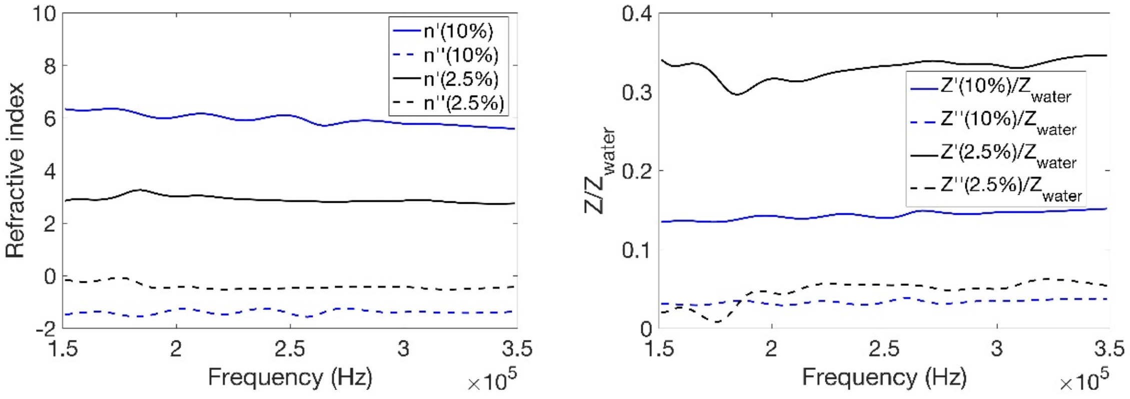

Supplementary Figure 2: Frequency measurements of the real (solid line) and imaginary (dotted line) parts of the refractive index (left panel) and impedance (right panel) for porous monodisk samples of porosity $\Phi = 2.5\%$ (black) and $\Phi = 10\%$ (blue).

Note that both samples are very weakly dispersive (a quasi constant speed of sound/acoustic index with frequency) over a wide frequency range of more than 200 kHz, thus ensuring broadband use of the metasurfaces with the same front-shaping function. As a result, the intrinsic attenuation n'' of the porous units has a minor impact on the overall quality of wavefront generation that relies more on the stability of n' with frequency. Similar to local impedance mismatch, the main effect of the porous rubber intrinsic absorption is a slight, variation in the amplitude along the phase fronts, right at the metasurface output. Nevertheless, the diffraction of the acoustic beam into the outer medium somehow balances the amplitude profile along the wavefronts after several wavelengths of propagation.
