# Supplementary material for: Flat acoustics with soft gradient-index metasurfaces
Source: Nat Commun. 2019 Jan 11;10:143. doi: 10.1038/s41467-018-07990-5 (PMC6329837; doi:10.1038/s41467-018-07990-5)
Supplement: Supplementary file 5 — Description of Additional Supplementary Files [file 41467_2018_7990_MOESM5_ESM.docx]

**Title:** Supplementary Movie 1
**Description:** Measured pressure field with no sample on the ultrasonic transducer

**Title:** Supplementary Movie 2
**Description:** Measured pressure field with the deflecting metasurface on the ultrasonic transducer

**Title:** Supplementary Movie 3
**Description:** Measured pressure field with the focusing metasurface on the ultrasonic transducer
